# THZ1 targeting CDK7 suppresses c-KIT transcriptional activity in gastrointestinal stromal tumours

**DOI:** 10.1186/s12964-022-00928-x

**Published:** 2022-09-08

**Authors:** Jianyi Sun, Qiang Zhang, Xiangfei Sun, Anwei Xue, Xiaodong Gao, Kuntang Shen

**Affiliations:** Department of General Surgery, Zhongshan Hospital, Fudan University School of Medicine, #180 Fenglin Road, Shanghai, 200032 China

**Keywords:** GIST, CDK7, THZ1, OSR1, Transcription regulation

## Abstract

**Background:**

Gastrointestinal stromal tumours (GISTs) are the most common mesenchymal tumours of the gastrointestinal tract and are characterized by activating mutations of c-KIT or PDGFRa receptor tyrosine kinases (RTKs). Despite the clinical success of tyrosine kinase inhibitors (TKIs), more than half of GIST patients develop resistance due to a second mutation. Cyclin-dependent kinase 7 (CDK7) is the catalytic subunit of CDK-activating kinase (CAK), and it plays an important role in the regulation of cell cycle transitions and gene transcription. THZ1, a CDK7 inhibitor, exhibits a dose-dependent inhibitory effect in various cancers.

**Methods:**

Data from the public GEO database and tissue microarray were used to analyse the gene expression levels of CDKs in GISTs. The impact of CDK7 knockdown and the CDK7 inhibitor THZ1 on GIST progression was investigated in vitro using CCK-8, colony formation, and flow cytometry assays and in vivo using a xenograft mouse model. RNA sequencing was performed to investigate the mechanism of GIST cell viability impairment mediated by THZ1 treatment.

**Results:**

Our study demonstrated that CDK7 is relatively overexpressed in high-risk GISTs and predicts a poor outcome. A low concentration of THZ1 exhibited a pronounced antineoplastic effect in GIST cells in vivo and in vitro. Moreover, THZ1 exerted synergistic anticancer effects with imatinib. THZ1 treatment resulted in transcriptional modulation by inhibiting the phosphorylation of Ser2, Ser5, and Ser7 within RNA polymerase II (RNAPII). c-KIT, an oncogene driver of GIST, was transcriptionally repressed by THZ1 treatment or CDK7 knockdown. Transcriptome sequencing analysis showed that OSR1 acts as a downstream target of CDK7 and regulates c-KIT expression. Taken together, our results highlight elevated CDK7 expression as a predictor of poor outcome in GIST and present the combination of CDK7 and RTK inhibitors as a potent therapeutic strategy to improve the efficacy of GIST treatment.

**Video abstract**

**Supplementary Information:**

The online version contains supplementary material available at 10.1186/s12964-022-00928-x.

## Introduction

Gastrointestinal stromal tumours (GISTs) originate from interstitial cells of Cajal (ICCs) and are the most common malignant mesenchymal neoplasms of the gastrointestinal tract [1, 2]. The majority of GISTs harbour activating mutations in *KIT* (75–80%) or platelet-derived growth factor receptor alpha (PDGFRa) (10–15%) [3–5]. The constitutive activation of these receptor tyrosine kinases (RTKs) in turn drives oncogenesis by activating downstream signalling pathways [6]. By inhibiting RTK signalling, tyrosine kinase inhibitors (TKIs) have been widely used as adjuvant therapy for GIST, and they significantly prolong survival [7–9]. However, despite initial responses, resistance to TKIs due to acquired secondary mutations limits their long-term benefit [10]. Nearly half of patients with advanced GIST show tumour progression within the first two years of imatinib treatment, and the estimated 10-year progression-free survival and overall survival rates are 7–9% and 19.4–23%, respectively [11, 12].

Several core transcription factors have been revealed to play essential roles in driving GIST cell proliferation and metastases by binding to enhancers of GIST-associated genes and facilitating *KIT* gene expression [13–15]. *FOXF1* is highly expressed in GISTs and colocalizes with *ETV1* at enhancers to directly control the transcription of two major oncogenes, *ETV1* and *KIT* [13]. Therefore, characterization of transcription factor deregulation in GIST may provide innovative insights into its pathogenesis mechanisms and offer new therapeutic approaches.

Cyclin-dependent kinases (CDKs) catalyse the phosphorylation of cyclins and control cell cycle transitions. CDK7 is the catalytic subunit of CDK-activating kinase (CAK), which can stimulate cell cycle progression and activate multiple other CDKs, such as CDK1, CDK2, CDK4, and CDK6, through T-loop phosphorylation [16–18]. In addition, CDK7 is a component of the transcription factor TFIIH, which phosphorylates the C-terminal domain (CTD) of RNA polymerase II (RNAP II) and therefore activates transcription initiation and elongation [19, 20]. CDK7 was found to be overexpressed and promote tumorigenesis in various cancers, such as breast cancer and osteosarcoma [21, 22]. THZ1, a selective CDK7 inhibitor, covalently binds to CDK7 and suppresses its kinase activity based on modification of a unique cysteine residue [23]. THZ1 potently represses the transcription of several superenhancer-associated oncogenes and elicits a tumour inhibition effect in various cancers [21, 24–28].

However, the role of CDK7 in the progression of GIST has not yet been elucidated. In this study, we found that CDK7 expression was elevated in high-risk GISTs and related to a poor prognosis. CDK7 knockdown or THZ1 treatment inhibits GIST cell proliferation and leads to inhibition of transcriptional activity and protein expression of *c-KIT.* Whole-transcriptome sequencing analysis was performed to decipher the mechanisms of CDK7 inhibition in GIST. THZ1 treatment led to inhibition of RNAPII phosphorylation, an indication of transcriptional inhibition, suggesting that targeting CDK7 may provide a mechanism to block transcriptional activation of *c-KIT* in GISTs. Taken together, our findings revealed that targeting CDK7 may be a potent therapeutic strategy for GIST patients.

## Methods

### Cell lines and reagents

The GIST-T1 cell line was purchased from Cosmo Bio Co. Ltd. (Tokyo, Japan). The GIST-882 cell line was kindly provided by Dr. Fletcher from Harvard Medical School. GIST-T1 and GIST-882 cells were cultured at 37 °C with 5% CO_2_ in RPMI-1640 medium (Gibco, USA) supplemented with 10% foetal bovine serum and 1% penicillin–streptomycin (Gibco, USA). Antibodies against CDK7 (#2916), Rpb1 CTD (#2629), phospho-Rpb1 CTD (Ser2) (#13499), phospho-Rpb1 CTD (Ser5) (#13523), phospho-Rpb1 CTD (Ser7) (#13780), cyclin D1 (#55506), CDK4 (#12790), γ(p-)H2AX (#9718), cleaved PARP (#5625), c-KIT (#3074), phospho-c-KIT (#3073), ERK1/2 (#4695), phospho-ERK1/2 (#4370), AKT (#9272), phospho-AKT (#4060), FLAG (#14793) and Alexa Fluor conjugated anti-rabbit IgG (H + L) (#8889) were purchased from Cell Signaling Technology (CST, MA, USA). An antibody against Ki67 (ab16667) was purchased from Abcam (Cambridge, MA, USA). An antibody against OSR1 (sc-376545) was purchased from Santa Cruz Biotechnology (Santa Cruz, CA). Antibodies against GAPDH (60004–1-Ig) and Caspase-3 (19677–1-AP) and HRP-conjugated secondary antibodies (SA00001-1 and SA00001-2) were purchased from Proteintech (Wuhan, China). Imatinib was kindly provided by Novartis Pharmaceuticals (Basel, Switzerland). THZ1 cells (HY-80013A) were purchased from MedChemExpress (MCE, Monmouth Junction, NJ, USA). Imatinib was dissolved in phosphate-buffered saline (PBS), and THZ1 was dissolved in dimethyl sulfoxide (DMSO) for the in vitro cell culture studies.

### Tissue microarray and immunohistochemistry

For immunohistochemistry (IHC), we employed a constructed tissue microarray (TMA) containing 181 paraffin-embedded primary GIST surgical samples resected at Zhongshan Hospital between 2009 and 2012 with Institutional Review Board approval. IHC staining was performed with anti-CDK7 antibody (#2916, CST). Two researchers separately evaluated the staining intensity and divided the samples into low expression and high expression groups.

### Cell transfection

Small interfering (si)RNAs targeting CDK7 and OSR1 and control siRNA were synthesized by Obio Technology (Shanghai, China). Plasmids and vector plasmids as controls were synthesized by Shanghai GeneChem Co., Ltd. siRNA and plasmid transfections were performed using Lipofectamine 2000 (Invitrogen) according to the manufacturer’s instructions. After transfection for 48 h, the knockdown efficiency was tested by western blotting. The sequences of siRNAs were listed in Additional file 1.

### Cell viability assay

Cell proliferation was examined using a Cell Counting Kit-8 (Yeasen, Shanghai, China) according to the instructions. GIST-T1 or GIST-882 cells were plated in 96-well plates at 1.5 × 10^3^ cells in 100 μl of medium per well. After incubation overnight, the medium was replaced with 100 µl medium with 10 μl CCK-8 and incubated for 2 h. The absorbance value was detected with a microplate reader at 450 nm. Similarly, the OD values were assessed at the indicated time points: 24, 48, and 72 h.

For the drug inhibition assay, 5 × 10^3^ GIST-T1 or GIST-882 cells/well were seeded and incubated at 37 °C overnight. Cells were treated with THZ1 and imatinib at various doses, and the mean inhibitory concentration (IC50) was calculated using nonlinear regression analysis in GraphPad Prism 8.0. The synergistic effect of the combination treatment was measured using CompuSyn software (ComboSyn, Inc. Paramus, NJ, USA) [29]. The combination index (CI) was generated by CompuSyn software, and CI < 1, = 1, and > 1 indicate synergic, additive or antagonistic effects, respectively.

### Cell cycle and apoptosis analysis

Approximately 1 × 10^6^ cells were collected and fixed with 75% ethanol at 4 °C overnight. After centrifugation and resuspension in PBS, the cells were incubated with 500 µL propidium iodide (PI)/RNase staining solution (Absin, China) at 37 °C for 30 min. Cell apoptosis was detected with an Annexin V-fluorescein isothiocyanate (FITC) apoptosis assay kit (Absin, China). Briefly, cells in 6-well plates were harvested by EDTA-free trypsinization, washed twice with cold PBS buffer and then resuspended in binding buffer with 5 µl of Annexin V and 5 µl of PI for 15 min at RT in the dark. The distribution of the cell cycle and apoptosis was determined in a BD Accuri C6 plus flow cytometer (BD Biosciences). The results were analysed by ModFit 3.0 software (Verity software house, Topsham, ME, USA) and FlowJo software (FlowJo, LLC, Ashland, OR, USA).

### Colony formation assay

The cells were seeded in 6-well plates at a density of 500 cells/well and cultured for 2 weeks. The medium was replaced every five days. To examine colony formation, the cells were fixed with 4% paraformaldehyde for 20 min. Next, crystal violet (0.5%) was used to stain the cells for 20 min. The colonies were photographed and then counted using ImageJ software.

### Quantitative real-time PCR (qRT–PCR)

Total RNA was extracted using TRIzol Reagent (Yeasen Biotechnology Shanghai, China) and reverse transcribed into cDNA using cDNA Synthesis SuperMix (Yeasen) according to the manufacturer’s instructions. Real-time PCR was performed using SYBR Green Master Mix (Yeasen) on a StepOne Real-Time PCR System (Applied Biosystems). The qRT-PCRs were run in triplicate. GAPDH was used as an endogenous control. The sequences of primers were listed in Additional file 1.

### Western blotting

Total protein was extracted from the cells with radioimmunoprecipitation assay (RIPA) buffer (Beyotime Biotechnology Shanghai, China) supplemented with protease inhibitors (Beyotime) and phosphatase inhibitors (Beyotime). The membranes were then blocked with 5% skim milk for 90 min at room temperature and incubated with the primary antibodies overnight at 4 °C. The blots were then washed 3 times for 10 min with TBST (TBS with 0.1% Tween 20) and incubated with HRP-conjugated goat anti-rabbit or mouse IgG secondary antibodies (1:5000) for 1 h at room temperature. The bands were visualized by incubating the blots with enhanced chemiluminescence (ECL, Yeasen) solution and were imaged by a Bio-Rad Imaging system detector.

### Immunofluorescence staining

Cells were treated with 100 nmol/L THZ1 for 24 h. Then, the cells were fixed with 4% paraformaldehyde for 10 min, permeabilized with 0.2% Triton X-100 (Beyotime) for 15 min, blocked with 3% BSA for 60 min, and incubated with anti-c-KIT antibody (1:400) overnight at 4 °C. The cells were incubated with Alexa Fluor conjugate-anti-rabbit IgG (H + L) secondary antibody (1:1000) for 60 min in the dark and then with DAPI (Yeasen) for 5 min.

### Mouse xenograft tumour assay

The mouse experiments were approved by the Institutional Review Boards of Zhongshan Hospital. Female BALB/c nude mice (4 weeks old) were obtained from Shanghai Jiesijie Laboratory Animal Co., Ltd. GIST-T1 cells were mixed with Matrigel (BD Biosciences) at a ratio of 1:1 and were subcutaneously injected into BALB/c nude mice at 3 × 10^6^ cells per mouse. When the tumours of all mice grew to be visible, the mice were randomly divided into two groups (n = 5). Mice were treated with THZ1 (10 mg/kg) or PBS intraperitoneally every three days. Tumour volume was measured every three days by using the formula: V = 1/2* length (mm) * width (mm)^2^. The mice were sacrificed when the tumour volume in the control group reached approximately 500 mm^3^. The tumours were harvested, weighed and used for IHC.

### Library preparation and sequencing

The RNA sequencing service was supplied by Applied Protein Technology (Shanghai, China). In brief, GIST-T1 cells were treated with DMSO or THZ1 (100 nmol/L) for 6 h. Then, RNA was extracted in triplicate by using TRIzol Reagent (Yeasen). Paired-end libraries were prepared using an ABclonal mRNA-seq Lib Prep Kit (ABclonal, China) following the manufacturer’s instructions. Sequencing was performed with an Illumina NovaSeq 6000/MGISEQ-T7 instrument. The ClusterProfiler R software package was used for Gene Ontology (GO) analysis [30]. When *p* < 0.05, the GO function was considered to be significantly enriched.

### Statistical analysis

All statistical analyses were performed with Prism 8.0 (GraphPad Software). Continuous variables are presented as the mean ± standard deviation (SD). A two-tailed Student’s t test was used to analyse the significance of differences between two groups, and one-way ANOVA was used for multiple groups. Statically significant differences were considered when* P* < 0.05.

## Results

### CDK7 mRNA and protein levels are elevated in high-risk GISTs and associated with poor clinical outcomes

**Fig. 1:**
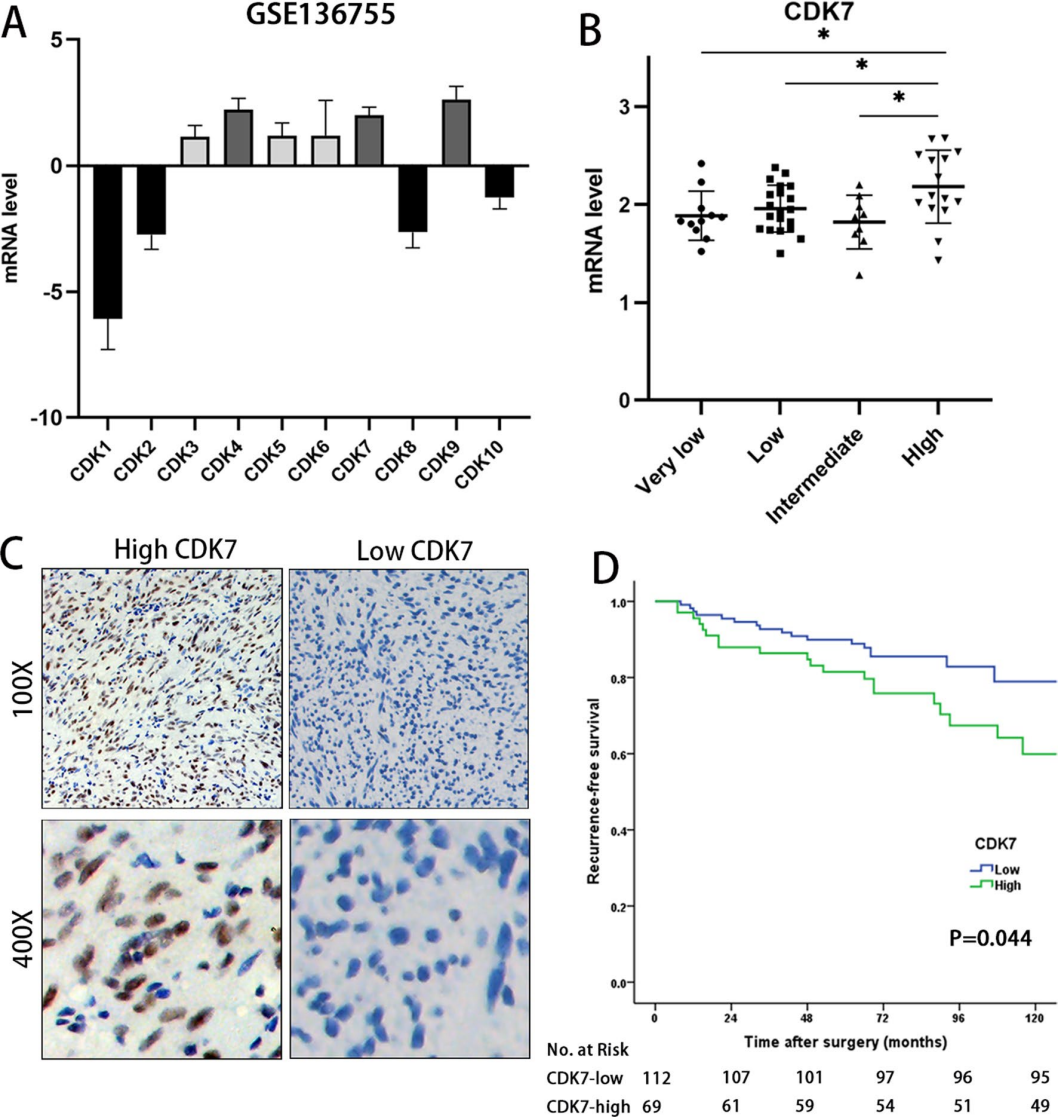
High CDK7 protein expression is associated with a poor prognosis of GIST. **A** Based on data from the GSE136755 dataset, the mRNA levels of CDK4, CDK7 and CDK9 were relatively higher than those of other CDKs for CDK1-10. The mRNA level was calculated via GEO2R. **B** CDK7 expression was significantly elevated in high-risk GISTs based on the data from GSE136755. **C** Representative scanned images of GIST samples with low or high CDK7 protein expression, as determined by IHC. **D** Kaplan–Meier survival curves with a risk table showing that high CDK7 protein expression was significantly positively related to poor recurrence-free survival in GIST patients (*P* = 0.044)

To analyse the differential CDK expression in GISTs, we obtained publicly available transcriptomic data of GSE136755 from the Gene Expression Omnibus (GEO), which includes gene microarray data and clinicopathological information for 59 primary GIST tumour samples without preoperative imatinib treatment [31]. The results showed that the mRNA levels of CDK4, CDK7 and CDK9 were relatively higher than those of the other CDKs for CDK1-CDK10 (Fig. 1A). Next, we measured the mRNA levels of CDK4, CDK7 and CDK9 in different GIST risk groups. CDK7 was significantly elevated in the high-risk group compared with the very low-, low- and intermediate-risk groups (*P* < 0.05 Fig. 1B), while the mRNA levels of CDK4 and CDK9 did not increase with the risk category (Additional file 1).

To further validate our findings at the protein level in GIST samples, we examined CDK7 protein expression in tissue microarrays (TMAs) of 181 GIST samples by immunohistochemistry (IHC). Two researchers separately evaluated the staining level and divided the patients into high and low CDK7 expression groups (Fig. 1C). We focused on the relationships between the expression level of CDK7 and clinicopathological characteristics, including sex, age, tumour size, mitotic index and risk category (Table 1). A significant correlation was found between elevated CDK7 expression, a higher mitotic index and a higher risk of GIST (*P* < 0.05), while no significant relationship was found between CDK7 expression and sex, age or tumour size. Kaplan–Meier survival analysis demonstrated that high CDK7 expression was strongly correlated with reduced recurrence-free survival (RFS) (*P* = 0.044, HR = 1.924, CI = 1.005–3.684, Fig. 1D).

**Table 1:** Relationship between CDK7 expression and clinicopathological characteristics of GIST patients

Factor	CDK7 expression		*P* value
	Low (112)	High (69)	
Age			
< 60	58	42	0.233
≥ 60	54	27	
Sex			
Male	51	36	0.385
Female	61	33	
Tumor site			
Stomach	74	41	0.374
Intestine, colorectal	34	27	
Others	4	1	
Tumor size (cm)			
0–5.0	63	35	0.725
5.1–10.0	39	26	
> 10	10	8	
Mitotic index (per 50 HPFs)*			
0–5	76	31	0.002
> 5	35	37	
Modified NIH criteria*			
Very low/low	48	18	0.011
Intermediate	22	9	
High	41	41	

*Two cases of mitotic index and modified NIH criteria were not accessible

In summary, CDK7 was highly expressed in high-risk GISTs and may participate in GIST progression.

### CDK7 knockdown attenuated GIST cell growth and induced cell cycle arrest

**Fig. 2:**
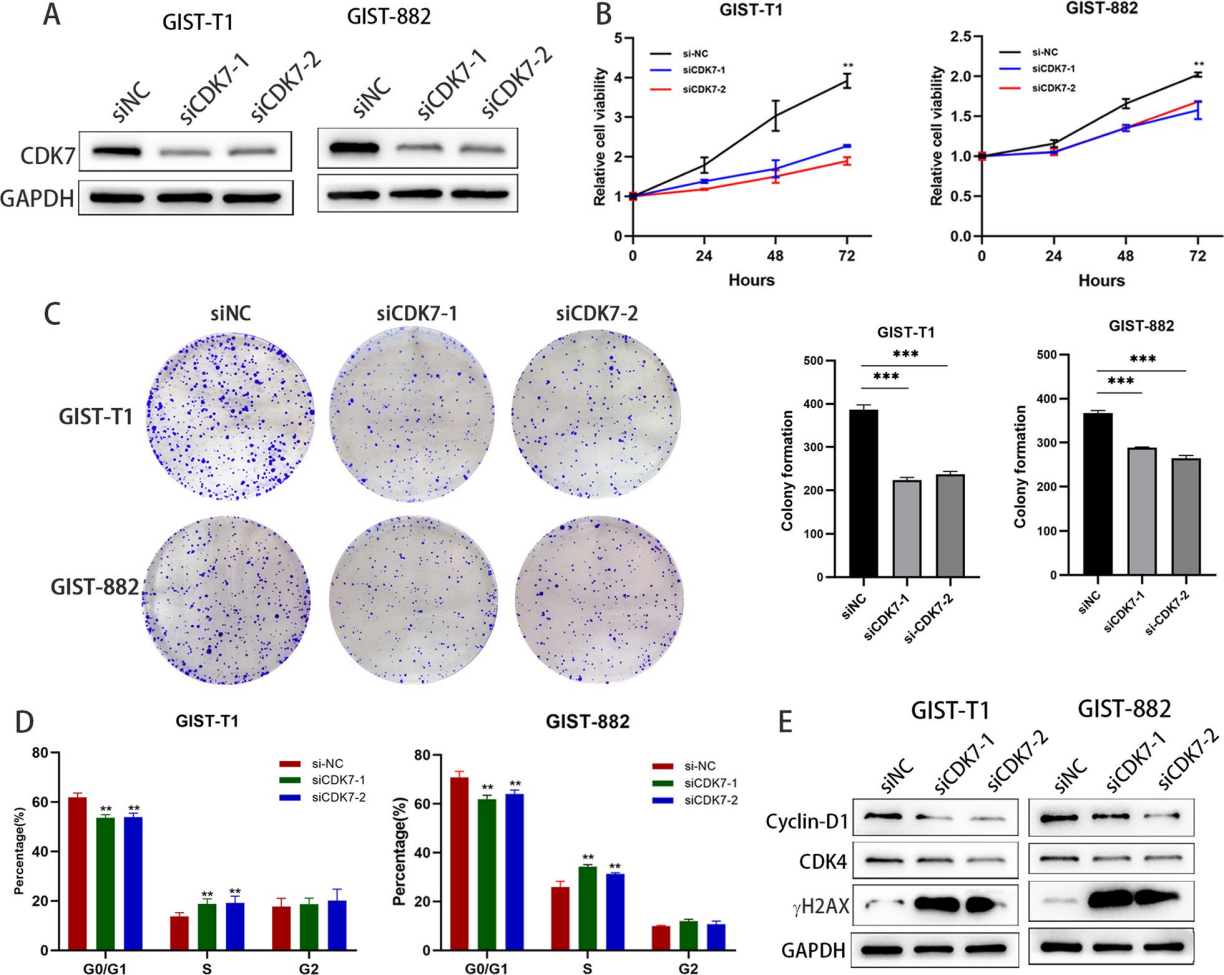
Knockdown of CDK7 decreases cell viability and proliferation and induces cell cycle arrest. **A** Immunoblotting analysis of CDK7 expression after targeting siRNA-mediated CDK7 knockdown in GIST-T1 and GIST-882 cells. A nontargeting siRNA and two independent siRNAs (siRNA1 and siRNA2) are represented by siNC, siCDK7-1, and siCDK7-2. **B** CCK-8 cell viability assay after CDK7 knockdown in GIST-T1 and GIST-882 cells. **C** Colony formation assays of GIST-T1 and GIST-882 cells after CDK7 knockdown. **D** Flow cytometry analysis was used to detect and analyse the cell cycle distribution after CDK7 knockdown. **E** Immunoblotting analysis of cyclin-D1, CDK4 and γH2AX expression after CDK7 knockdown

To investigate the role of CDK7 in GIST cells, two independent siRNAs were transfected into GIST-T1 and GIST-882 cells. The transfection efficacy was detected by western blotting (Fig. 2A). As shown in Fig. 2B and C, CDK7 knockdown significantly suppressed cell proliferation and colony formation in both GIST T1 and GIST-882 cells. We next assessed the effect of CDK7 knockdown on GIST cell cycle progression. We found that CDK7 knockdown resulted in marked cell cycle arrest at the G1/S phase (Fig. 2D). These results indicate that CDK7 may play an oncogenic role in GIST cells. We also found that the cell cycle-related protein cyclin D1, a regulator of the G0–G1 to S-phase transition, was significantly suppressed. Additionally, γH2AX was significantly upregulated, suggesting increased DNA damage after CDK7 knockdown (Fig. 2E). Taken together, these data demonstrated that CDK7 might be a promising treatment target for GISTs.

### THZ1 exerted dose-dependent tumour inhibition and induced apoptosis in GIST cells

**Fig. 3:**
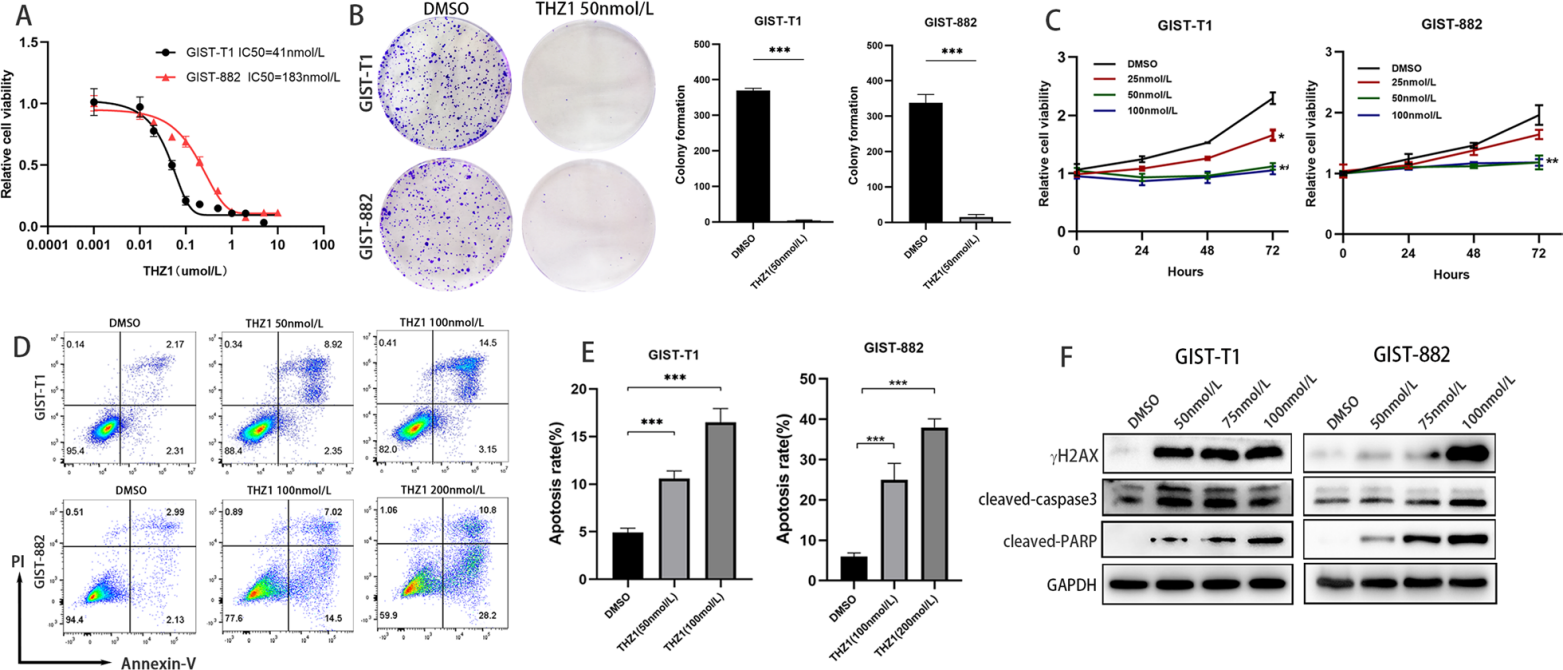
THZ1 suppresses the proliferation and induces the apoptosis of GIST cells in vitro. **A** Dose–response curves of GIST-T1 and GIST-882 cells after treatment with THZ1 for 72 h and 144 h, respectively. Cell viability was assessed with the CCK-8 assay. **B** Colony formation assays of GIST-T1 and GIST-882 cells treated with THZ1. **C** Time-response curves of GIST-T1 and GIST-882 cells upon treatment with THZ1 at concentrations as low as 25, 50, and 100 nmol/L. **D** Flow cytometry analysis in GIST cells treated with THZ1 at the indicated concentrations or vehicle. **E** Immunoblotting analysis of cleaved caspase 3 and cleaved PARP protein expression after THZ1 treatment for 24 h

The CDK7 inhibitor THZ1 exerts antitumorigenic effects in various cancers. To evaluate the antitumour effects of THZ1 in GIST, GIST-T1 and GIST-882 cells were treated with THZ1 for 72 h and 144 h, respectively. As shown in Fig. 3A and B, THZ1 significantly suppressed the viability of GIST cells in a dose-dependent manner. The IC50 values were 41 nmol/L and 183 nmol/L for GIST-T1 and GIST-882 cells, respectively. At concentrations as low as 25, 50, or 100 nmol/L, THZ1 potently reduced the viability of GIST-T1 and GIST-882 cells in a time-dependent manner (Fig. 3C).

The extent of apoptosis was assessed after THZ1 treatment with flow cytometry analysis. Annexin V/PI staining illustrated that the percentage of apoptotic cells significantly increased in a THZ1 dose-dependent manner (Fig. 3D, E). Apoptosis-related proteins were also detected. As shown in Fig. 3F, the expression of cleaved PARP, cleaved caspase-3 and γH2AX was markedly increased in GIST-T1 and GIST-882 cells after THZ1 treatment.

Collectively, these results revealed that THZ1 could inhibit GIST cell proliferation and induce apoptosis.

### THZ1 showed antineoplastic properties in vivo

**Fig. 4:**
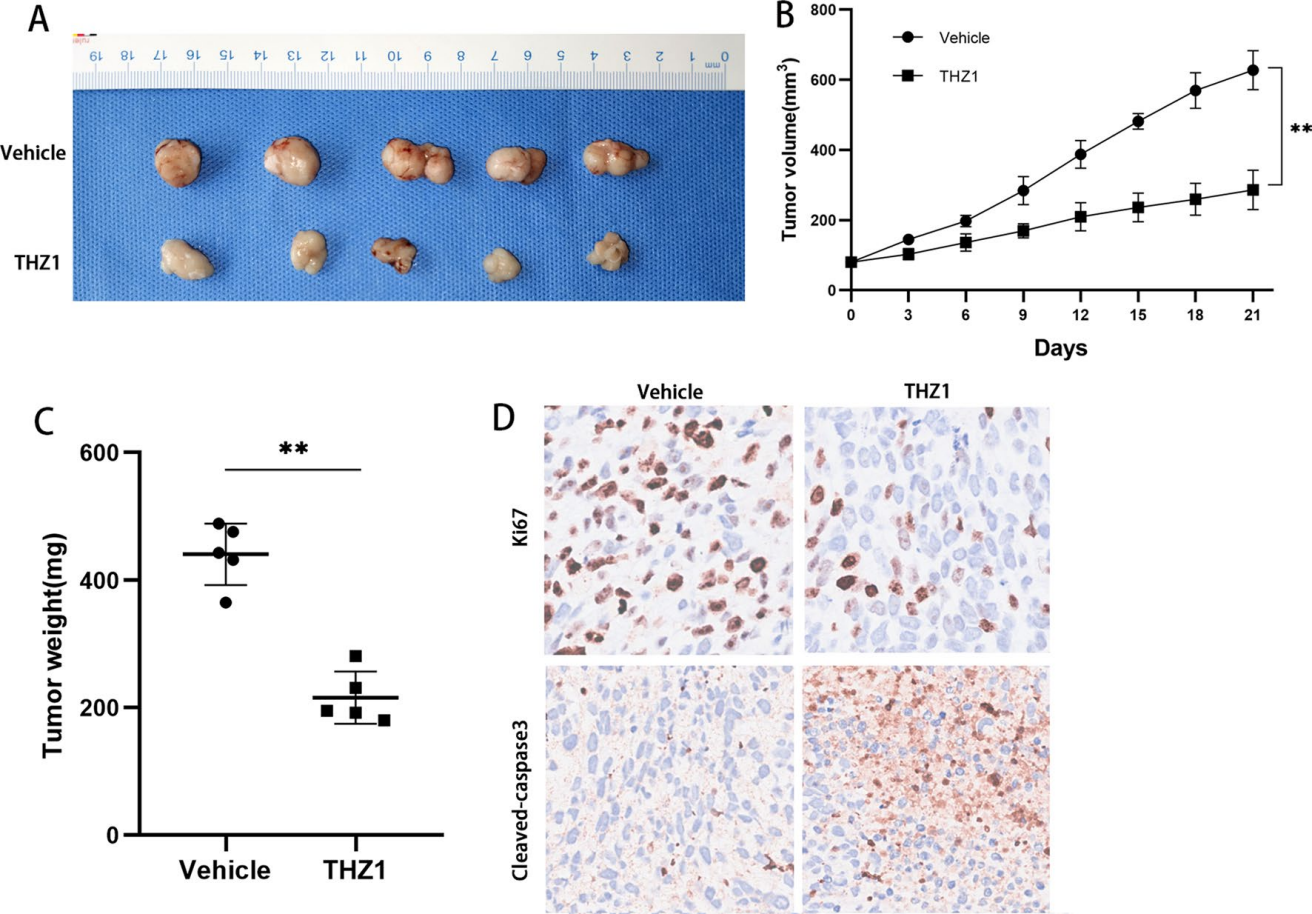
THZ1 shows antineoplastic properties in vivo. **A** Images of subcutaneous tumours from the vehicle or THZ1 treatment groups (n = 5). **B**, **C**. Tumour volume and weight of the subcutaneous tumour model. THZ1 treatment led to a significant reduction in tumour volume and weight. **C** Immunohistochemistry staining of Ki67 and cleaved caspase-3 in tissue sections from vehicle- or THZ1-treated subcutaneous tumours

The tumour inhibitory effect of THZ1 in vivo was assessed in a subcutaneous xenograft model of GIST-T1. As expected, the results showed that THZ1 treatment led to a profound reduction in tumour volume and weight, corroborating the tumour inhibition effect in vitro (Fig. 4A). Notably, THZ1 treatment resulted in a significant reduction in tumour volume and weight (Fig. 4B, C). Ki67 and cleaved-caspase 3 immunostaining assays of tumour samples showed that THZ1 treatment dramatically inhibited cell proliferation and promoted cell apoptosis (Fig. 4D). Collectively, these results revealed that THZ1 exerts a potent antitumor effect on GIST cells in vivo.

### THZ1 exhibited synergistic inhibition with imatinib in GIST cells

**Fig. 5:**
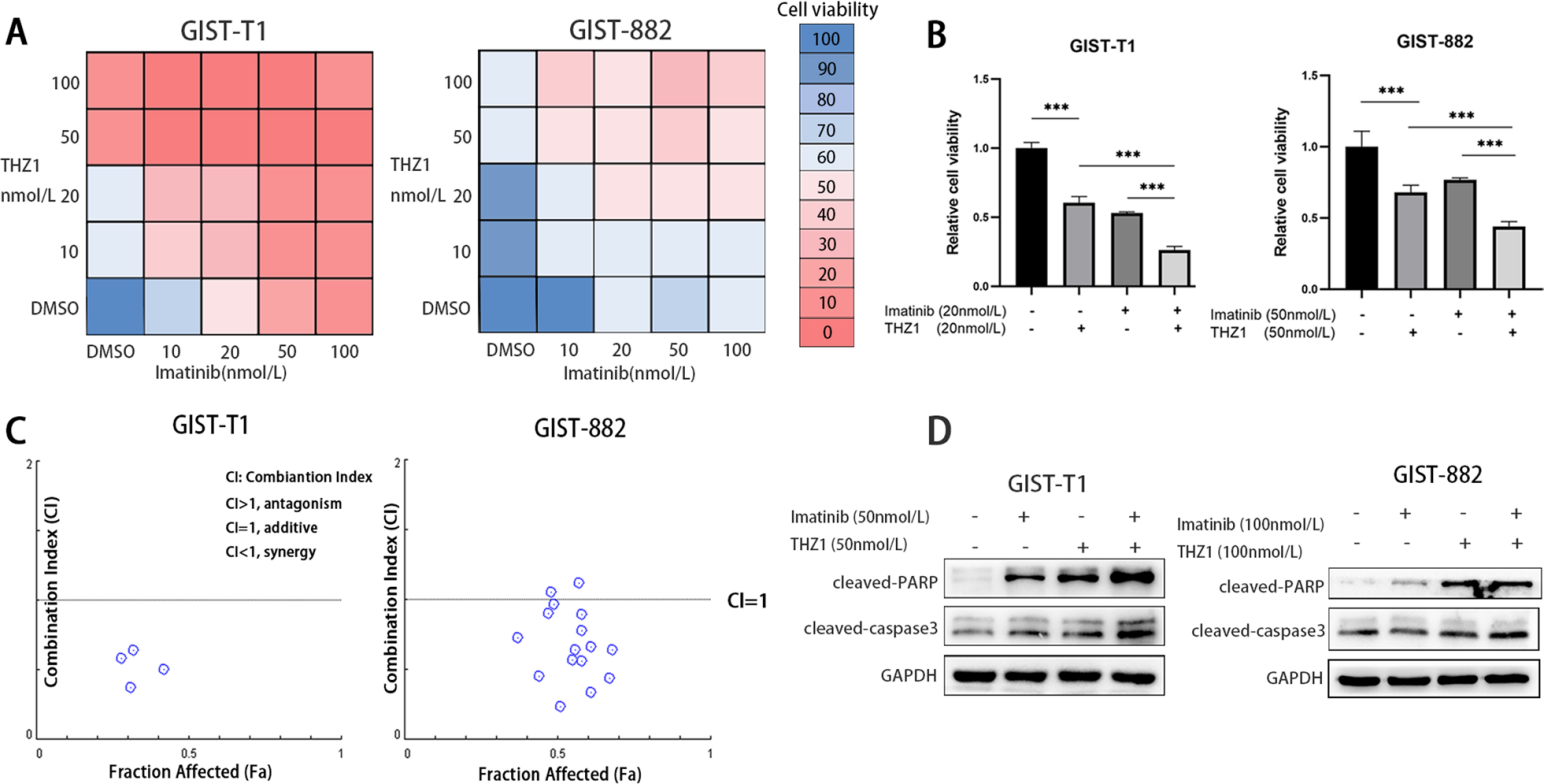
Combination treatment with THZ1 and imatinib for GIST. **A** CCK-8 viability assay following treatment for 72 h with escalating concentrations of THZ1 and imatinib in GIST-T1 and GIST-882 cells. **B** GIST-T1 cells received single 20 nmol/L imatinib, THZ1 and a combination treatment for 72 h. GIST-882 cells received single 50 nmol/L imatinib, THZ1 and a combination treatment for 72 h. The cell viability of combination treatment group was significantly suppressed than monotherapy. **C** Synergy was calculated using CompuSyn, and a combination index value under 1.0 was considered to indicate synergy. **D** Immunoblotting analysis of cleaved caspase 3 and cleaved PARP protein expression after combination treatment with THZ1 and imatinib in GIST-T1 and GIST-882 cells for 24 h

Furthermore, we assessed the potential tumour inhibition efficacy of THZ1 in combination with imatinib in GIST-T1 and GIST-882 cells. Cells were treated with a series of different drug concentrations. The synergistic antitumour effect was analysed by using CompuSyn software, and combination index (CI) values were calculated based on the drug combination principles proposed by Chou-Talalay [29]. Our data revealed that single treatment with THZ1 or imatinib significantly suppressed GIST-T1 and GIST-882 cell viability (Fig. 5A). THZ1 had strong synergistic effects with imatinib in both GIST-T1 and GIST-882 cells, and there was an advantage of combination treatment (Fig. 5A–C). Immunoblotting analyses showed that combination treatment with THZ1 and imatinib led to more upregulation of cleaved caspase 3 and PARP than single agent treatment (Fig. 5D). In summary, combination treatment with THZ1 and imatinib significantly enhanced the GIST cell viability inhibition effect.

### THZ1 treatment caused selective transcription suppression in GIST

**Fig. 6:**
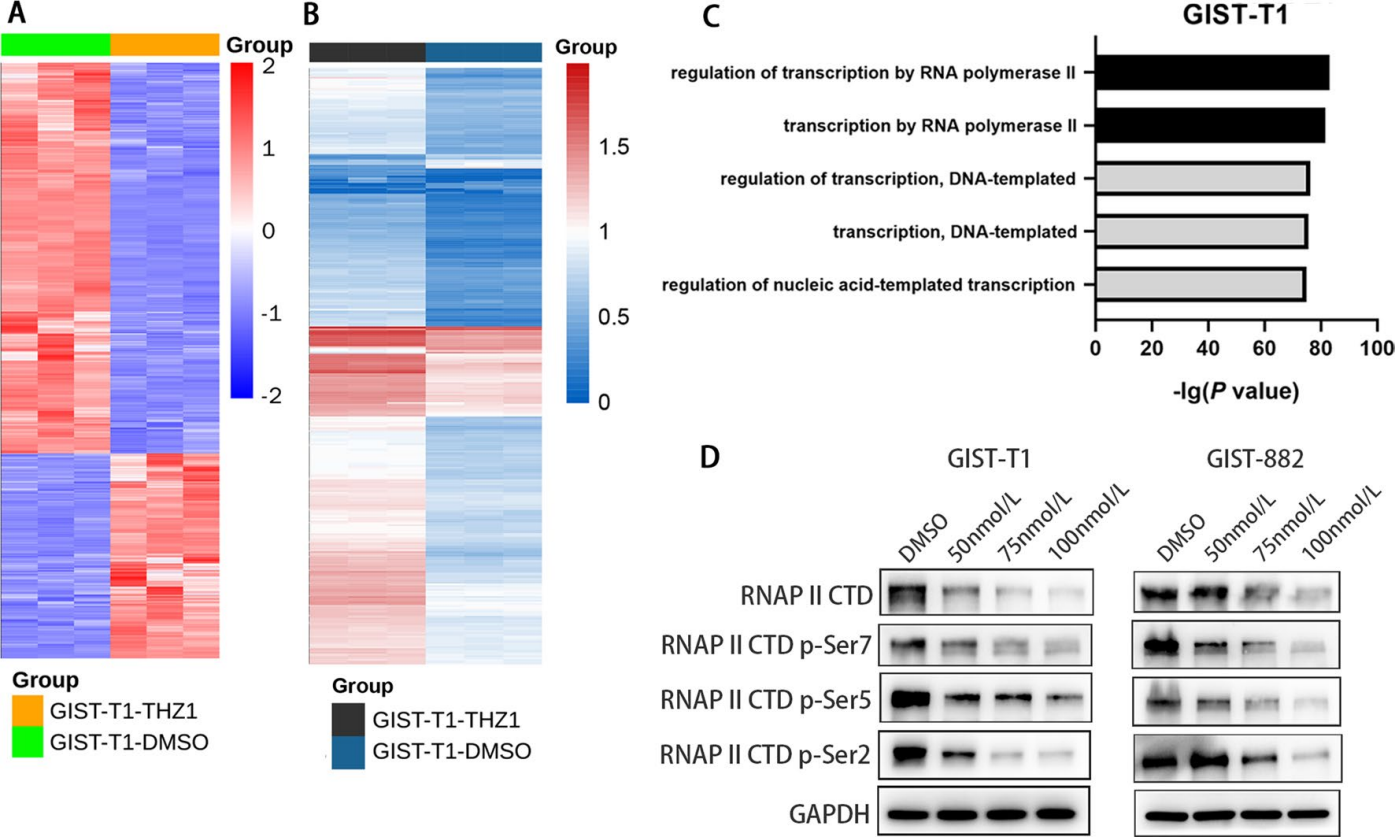
CDK7 inhibition leads to the suppression of RNA transcription in GIST cells. **A** Heatmap showing the change in global active transcripts in GIST-T1 cells following treatment with 50 nmol THZ1 and DMSO for 6 h. **B** Heatmap showing the transcriptional changes in cell cycle-related genes from the gene set (GOBP_CELL_CYCLE.v7.5.1) in GIST-T1 cells after THZ1 treatment. **C** The enriched GO functional terms of the transcripts that were reduced over twofold in GIST-T1 cells following treatment with 50 nmol/L THZ1 for 6 h. **D** Immunoblot analyses of RNAPII, RNAPII CTD phosphorylation (S2, S5, and S7), and CDK7 in GIST-T1 and GIST-882 cells treated either with THZ1 or DMSO at the indicated concentrations for 24 h

We next probed the mechanisms underlying the antitumour effect of THZ1 in GISTs. Considering the prominent role of CDK7 in regulating the cell cycle and RNAPII-mediated transcription, we next assessed THZ1-induced transcriptional alterations in the gene expression profiles of GIST cells by whole-transcriptome sequencing (RNA-sequencing, RNA-seq) analyses. Treatment with 50 nmol/L THZ1 for 6 h resulted in a dramatic decrease in global messenger RNA levels (Fig. 6A). A gene set (GOBP_CELL_CYCLE.v7.5.1) was downloaded and analysed with our RNA-seq data. Among 1749 genes, 620 genes were significantly downregulated after THZ1 treatment (*P* < 0.05, log2-fold change > 1), and 320 genes were significantly upregulated (Fig. 6B). We performed gene ontology (GO) analysis to explore the functional enrichment of most differentially expressed genes. We found that THZ1-sensitive genes were associated with pathways involved in the regulation of transcription regulation by RNAPII (Fig. 6C). Because CDK7 can preferentially downregulate RNAPII CTD phosphorylation, we detected the related proteins by western blotting. THZ1 treatment reduced the phosphorylation of Ser2, Ser5 and Ser7 on the Pol II CTD in GIST-T1 and GIST-882 cell lines in a dose-dependent manner (Fig. 6D). Although our results showed that CDK7 knockdown led to cell cycle arrest in GIST cells, we found that CDK7 preferentially regulated transcription instead of directly regulating the cell cycle in GIST.

### CDK7 knockdown and THZ1 treatment inhibited c-KIT transcription

**Fig. 7:**
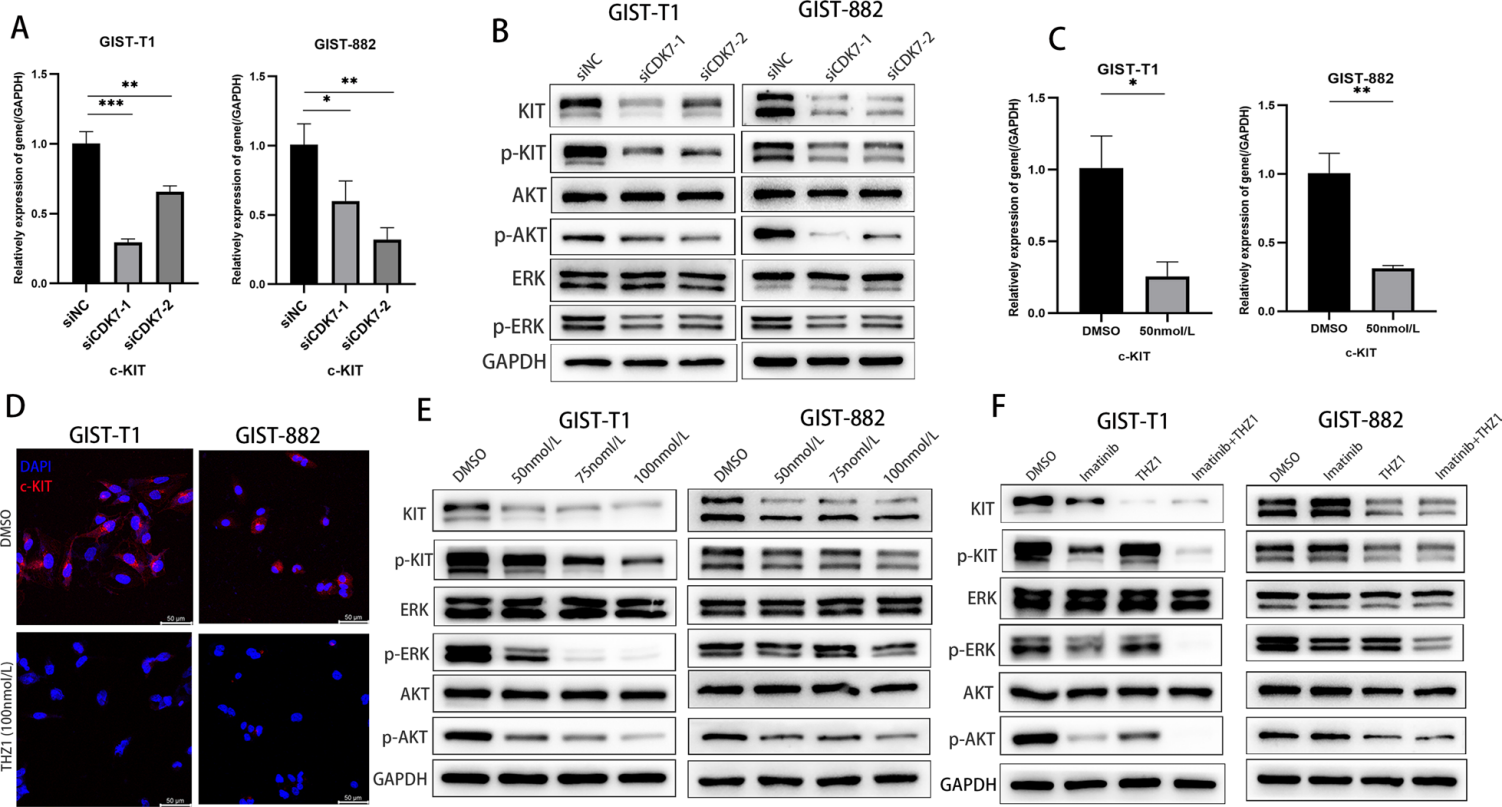
CDK7 inhibition leads to the suppression of c-KIT transcriptional activity and protein expression in GIST cells. **A, B** qRT–PCR and immunoblotting analysis of c-KIT expression after siRNA-mediated CDK7 knockdown in GIST-T1 and GIST-882 cells. **C**, **D** qRT–PCR and immunofluorescence assays showed that c-KIT expression changed after THZ1 treatment in GIST-T1 and GIST-882 cells. **E** Immunoblotting analysis showed that the expression of c-KIT and downstream ERK and AKT signalling pathway components was inhibited following THZ1 treatment at the indicated concentration in GIST-T1 and GIST-882 cells for 24 h. **F** Immunoblotting analysis indicated that combination treatment with imatinib and THZ1 enhanced c-KIT expression inhibition in GIST-T1 and GIST-882 cells for 24 h

Previous research revealed that THZ1 treatment induces EGFR and PDGFRa expression and multiple downstream oncogenic signalling pathways in glioma [32]. CDK7 inhibition suppresses GIST cell proliferation and survival via inhibition of transcription. Therefore, we analysed the c-KIT expression changes after CDK7 knockdown or THZ1 treatment. Interestingly, we observed that c-KIT mRNA and protein levels were significantly decreased after CDK7 knockdown (Fig. 7A, B) and THZ1 treatment (Fig. 7C–E). The phosphorylated forms of AKT and ERK were all prominently downregulated by CDK7 or THZ1 knockdown (Fig. 7B, E). Compared with monotherapy with imatinib or THZ1, the combination of the two more effectively inactivated the c-KIT and downstream AKT and ERK signalling cascades (Fig. 7F).

### CDK7 inhibited c-KIT transcription via OSR1 in GIST cells

**Fig. 8:**
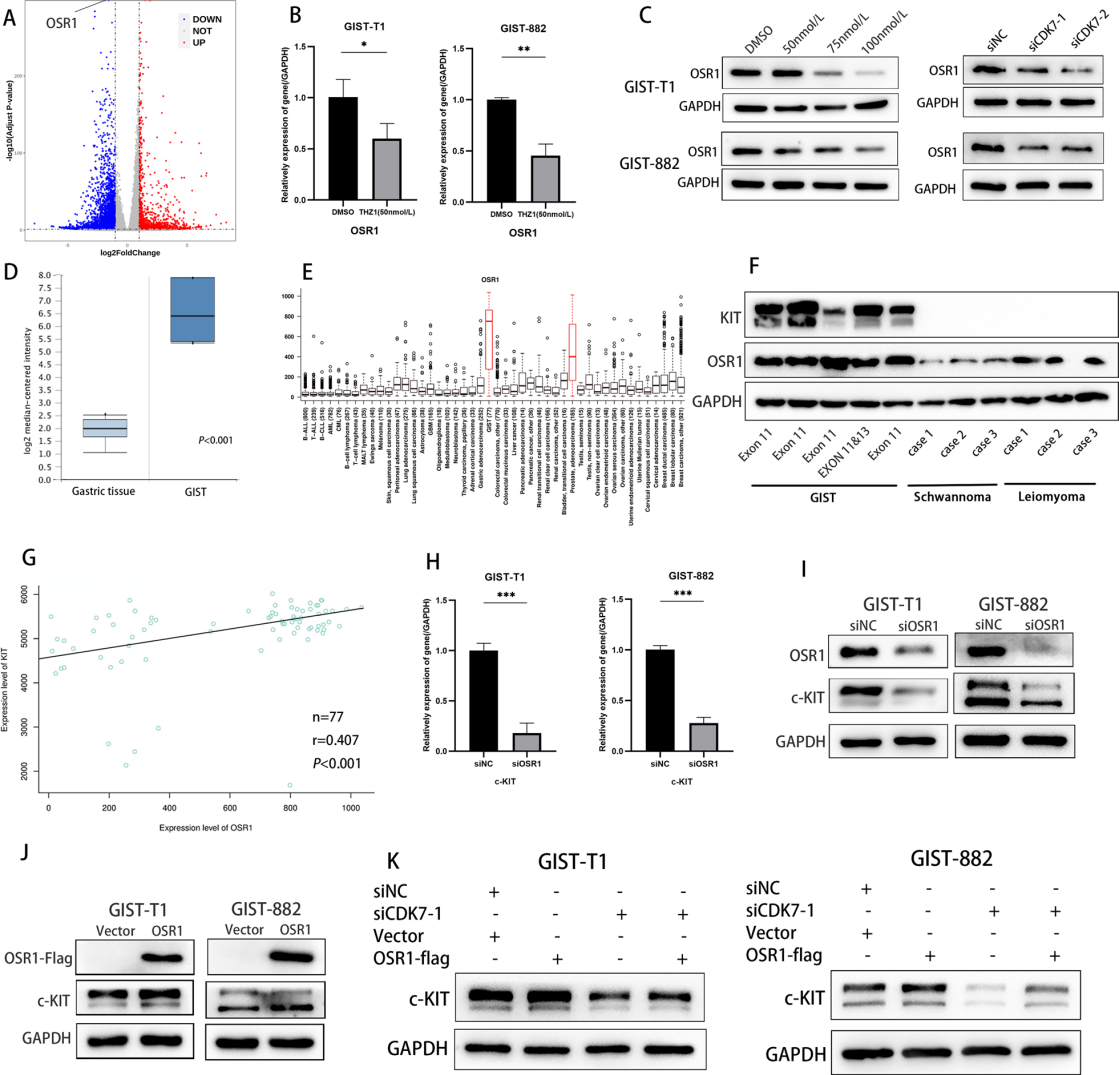
CDK7 inhibits c-KIT expression through OSR1 in GIST. **A** Volcano plot of RNA-seq data displaying the distribution of differential gene expression between THZ1 treatment and DMSO treatment. The upregulated and downregulated genes are highlighted in red and blue, respectively. The results illustrated that OSR1 was the top downregulated gene with the lowest *P* value. **B**, **C** qRT–PCR and immunoblotting analyses showed that OSR1 expression was inhibited by CDK7 knockdown or THZ1 treatment in a dose-dependent manner. OSR1 expression was inhibited by CDK7 knockdown in GIST-T1 and GIST-882 cells. **D** Gene expression profile in the Oncomine database. The results showed that OSR1 was significantly upregulated in GIST compared with normal gastric tissue. **E** Gene expression profile in the MediSapiens IST Online transcriptome database. The results showed that OSR1 was uniquely overexpressed in GIST and prostate cancer compared with other cancer types. F Immunoblot analyses of OSR1 expression in clinical sample tissues of GIST, gastrointestinal leiomyoma and schwannoma. The expression of OSR1 was significantly higher in GIST than in leiomyoma and schwannoma. **G** Data from the MediSapiens database showed that c-KIT and OSR1 expression levels were positively correlated (n = 77, r = 0.407, *P* < 0.001). **H**, **I** qRT–PCR and immunoblotting analyses showed that OSR1 knockdown significantly inhibited c-KIT mRNA and protein expression in GIST-T1 and GIST-882 cells. **J** Immunoblot analyses showed that OSR1 overexpression promoted c-KIT expression in GIST-T1 and GIST-882 cells. **K** The inhibitory effect of c-KIT expression was reversed when CDK7 siRNA and OSR1 plasmid were cotransfected into GIST-T1 and GIST-882 cells

Previous studies have indicated that THZ1 impairs the transcriptional activity of the superenhancers (SEs) of oncogenic genes, so to explore the mechanism underlying c-KIT transcription inhibition induced by CDK7 knockdown, we investigated SEs. Previous research has identified a cluster of SEs of GISTs, including MEIS1, OSR1, and FOXF1. Interestingly, we found that odd-skipped related transcription factor 1 (OSR1) was the most significantly downregulated gene in GIST-T1 cells after THZ1 treatment (Fig. 8A, Additional file 2: Table S1). Immunoblotting analyses verified that OSR1 expression was significantly inhibited by THZ1 treatment with CDK7 knockdown (Fig. 8B, C). Next, we searched the gene expression profile in the Oncomine database and MediSapiens IST Online transcriptome database. As shown in Fig. 8D, E, OSR1 was significantly upregulated in GIST compared with normal gastric tissue and was highly expressed in GIST and prostate cancer compared with other human cancers. To verify this result, we detected OSR1 expression in clinical samples of GIST, gastrointestinal leiomyoma and schwannoma. As expected, OSR1 was significantly highly expressed in GIST (Fig. 8F). Furthermore, we performed correlation analysis of OSR1 and c-KIT expression, and the results showed that OSR1 levels were positively associated with c-KIT levels (r = 0.407, *P* < 0.001, Fig. 8G). The qRT–PCR and immunoblotting analyses showed that OSR1 knockdown significantly inhibited c-KIT transcript and protein expression in GIST-T1 and GIST-882 cells (Fig. 8H, I). In contrast, overexpressed OSR1 increased c-KIT expression (Fig. 8J). When CDK7 siRNA and OSR1 plasmid were cotransfected into GIST-T1 and GIST-882 cells, the inhibition of c-KIT expression was reversed (Fig. 8K). Collectively, these results revealed that CDK7 mediates c-KIT expression through OSR1.

## Discussion

Despite advances in genetic alteration diagnoses and tyrosine kinase inhibitor treatment, the prognosis of patients with GIST remains unsatisfactory, especially for high-risk GISTs. The effectiveness of standard targeted therapy is hampered by secondary resistance following initial responses due to acquired secondary mutations [10].

CDKs play a significant role in regulating the cell cycle and gene transcription. CDK7 is one of the subunits of the multiprotein transcription factor complex TFIIH, and it is critical for facilitating transcription initiation and elongation via phosphorylation of the CTD of RNAPII [19, 20]. CDK7 has been reported to be a potential therapeutic target in transcription-dependent cancers and is associated with a poor prognosis [21, 24–28]. However, the role of CDK7 in GIST tumorigenesis remains unknown.

In our study, we conducted an analysis of the transcriptomic data of 65 GIST patients from the GEO public database. The CDK4, CDK7 and CDK9 mRNA levels were significantly higher than those of other CDKs, and further, we found that the CDK7 mRNA level was significantly elevated in high-risk GISTs, while there was no significant difference in CDK4 and CDK9. Previously, Yu Liu analysed quantitative proteome profiling of GIST and adjacent normal tissue and found that several kinases were significantly upregulated in GIST, including KIT and CDK7 [33]. Consistent with this, we validated with tissue microarrays (TMAs) that CDK7 overexpression in GISTs was correlated with tumour progression and an unfavourable prognosis. Inhibition of CDK7 or CDK7 inhibitor treatment significantly inhibited GIST cell proliferation. These results revealed that CDK7 might play an oncogenic role in GIST progression.

GISTs exhibit a homogeneous repertoire of transcription factors, which supports its associated gene expression program throughout all stages of the disease. Several core transcription factors have been revealed to play essential roles in driving GIST cell proliferation and metastasis by binding to enhancers of GIST-associated genes and facilitating *KIT* gene expression [13–15]. Moreover, a previous study revealed that the KIT-regulated enhancer domain in GISTs could be targeted by BRD4, a key activator of RNAPII transcription at active chromatin marks, and the BET bromodomain inhibitor (BBI) can downregulate *KIT* transcription [34, 35]. Therefore, characterization of transcription factor deregulation in GIST may provide innovative insights into the pathogenesis mechanisms and offer new therapeutic approaches.

In our study, RNA-seq analysis was used to detect alterations in total transcripts in GIST cells after treatment with THZ1. We observed that a cluster of genes was particularly sensitive to THZ1 treatment and was mainly enriched in biological processes of transcription regulation mediated by RNAPII. Considering the original genetic alteration of GIST, we investigated the transcriptional activity and protein expression of c-KIT after CDK7 knockdown or THZ1 treatment. Interestingly, we found that c-KIT transcription and protein expression were significantly inhibited after CDK7 knockdown or THZ1 treatment in both GIST T1 and 882 cells. This result indicated that CDK7 might be a key driver of c-KIT expression in GIST and that it is a possible therapeutic target.

CDK inhibitors are of great interest as novel therapeutic agents against cancer, and several CDK inhibitors have been applied in the clinic. CDK4/6 inhibitors have gained FDA approval for the treatment of hormone receptor-positive breast cancer, and inhibitors targeting other cell cycle CDKs are currently in clinical trials for non-small cell lung cancer and other solid tumours [36]. THZ1, a CDK7 inhibitor, exerts synergistic anticancer effects when combined with TKIs against neuroblastoma, glioma and non-small cell lung cancer [32, 37–39]. Therefore, targeting CDK7 may provide an alternative therapeutic option to block the reactivation of receptor tyrosine kinase pathways in RTK-driven neoplasms, especially for TKI-resistant cancer. Our research revealed that a combination of THZ1 and imatinib exerts synergistic antitumour effects in GIST imatinib-sensitive cells. To investigate the impact of CDK7 on c-KIT expression in GIST cells and the synergistic effect of THZ1 and imatinib, GIST-T1 and GIST-882 cells, which both harbour c-KIT gene mutations and are sensitive to imatinib, were used in our research. Both THZ1 and imatinib treatment led to c-KIT expression inhibition, and the combination treatment enhanced the inhibition of c-KIT expression and the downstream AKT and ERK signalling pathways. Taken together, our results indicate that THZ1 has effective antitumour activity against GISTs and may provide an additional therapeutic strategy for GIST patients with a poor response to imatinib.

A superenhancer (SE) is a large cluster of genomic regulatory elements typically exhibiting an enrichment of histone H3 lysine 27 (H3K27ac) and densely bound by transcription factors and cofactors, playing critical roles in defining cell fate and identity [40]. Interestingly, superenhancers frequently drive the expression of prominent oncogenes in cancer cells [41]. Previous research has used H3K27ac chromatin immunoprecipitation with sequencing (ChIP-seq) of GIST tumour samples and cell lines and identified the SE clusters that drive c-KIT gene expression and are unique to GISTs [15]. Subsequently, studies indicated that the SE domain was essential for c-KIT gene expression and tumorigenesis, including FOXF1, HAND1 and BARX1 [13, 14]. Disruption of the SE domain represents a therapeutic vulnerability in GIST [34]. Among the genes screened out by ChIP-seq of GIST tumour samples and cell lines, OSR1 was hypothesized to bind to the c-KIT locus by ATAC sequencing [15]. In our research, we found that CDK7 knockdown significantly inhibited the expression of c-KIT, and we identified the genes downregulated after THZ1 treatment by RNA-seq. Interestingly, we found that OSR1 was the predominantly downregulated gene. Therefore, we hypothesized that CDK7 knockdown inhibited the expression of c-KIT via OSR1. Subsequently, we proved that OSR1 expression was inhibited by THZ1 treatment in a dose-dependent manner and that OSR1 knockdown also significantly inhibited c-KIT expression. Moreover, OSR1 overexpression reversed the inhibition of c-KIT expression induced by CDK7 knockdown. In summary, our research may have revealed the role of OSR1 in c-KIT expression attenuated by CDK7 inhibition.

In summary, our results uncovered the positive correlations between CDK7 and the malignant potential of GISTs and indicated that targeting CDK7 with the selective inhibitor THZ1 may be a promising treatment for GIST patients.

## Supplementary Information

**Additional file 1**. The siRNA sequences and qRT-PCR primer sequences used in this research..

**Additional file 2**. The different expression gene set of GIST-T1 cells after THZ1 treatment by RNA-sequencing analysis.

## Data Availability

All data supporting the conclusions within the article are included in the additional file and are available from the corresponding author at shen.kuntang@zs-hospital.sh.cn.
