# Supplementary material for: THZ1 targeting CDK7 suppresses c-KIT transcriptional activity in gastrointestinal stromal tumours
Source: Cell Commun Signal. 2022 Sep 8;20:138. doi: 10.1186/s12964-022-00928-x (PMC9454178; doi:10.1186/s12964-022-00928-x)
Supplement: Supplementary file 2 — Additional file 1. The siRNA sequences and qRT-PCR primer sequences used in this research.. [file 12964_2022_928_MOESM2_ESM.docx]

**Corresponding author**

Kuntang Shen, Department of General Surgery, Zhongshan Hospital, Fudan University School of Medicine, #180 Fenglin Road, Shanghai 200032, China. E-mail: [shen.kuntang@zs-hospital.sh.cn](mailto:shen.kuntang@zs-hospital.sh.cn).

Xiaodong Gao, Department of General Surgery, Zhongshan Hospital, Fudan University School of Medicine, #180 Fenglin Road, Shanghai 200032, China. E-mail: <gao.xiaodong1@zs-hospital.sh.cn>.


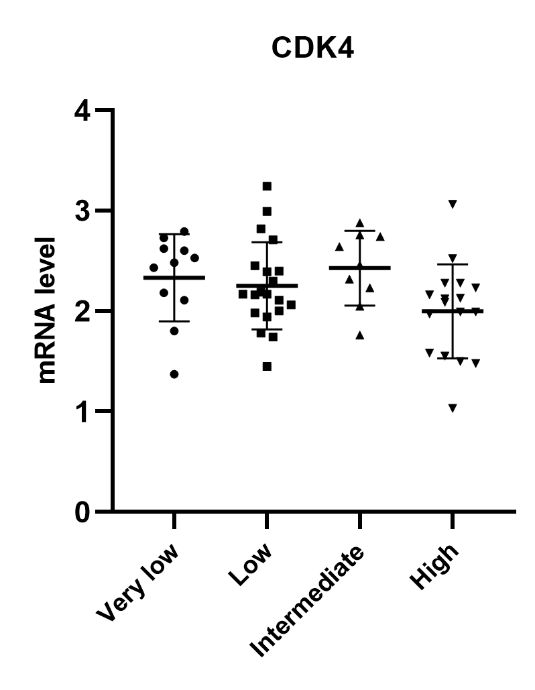

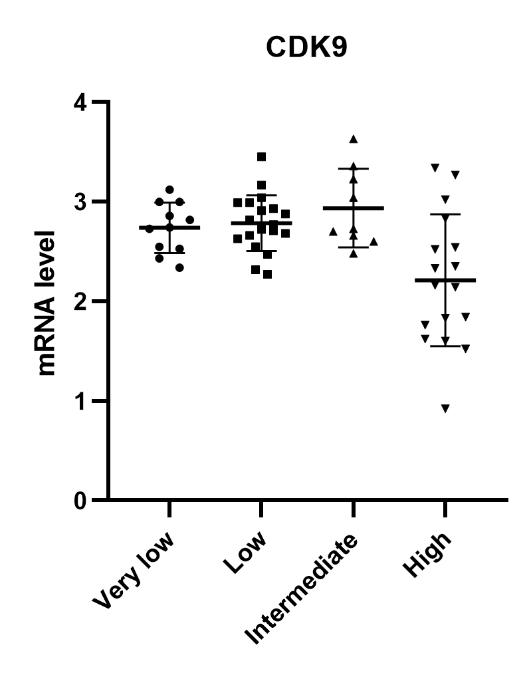


Fig 1. The mRNA levels of CDK4 and CDK9 did not increase with the risk category

**siRNA sequences**

The sequences of CDK7 siRNA are listed as follows: siCDK7#1, 5′-GGACAUAAAUCUAAUAUUATT-3′, 3′-UAAUAUUAGAUUUAUGUCCTT-5′; siCDK7#2, 5′-GGACAUAGAUCAGAAGCUATT-3′, 3′-UAGCUUCUGAUCUAUGUCCTT-5′. siOSR1, 5′-CCUCCAAGAUCAAAUGCUATT-3′, 3′-UAGCAUUUGAUCUUGGAGGTT-5′.

**qRT-PCR** [**primer**](javascript:;) **sequence**

For qRT-PCR, the sense primer for c-KIT was 5’-CGTTCTGCTCCTACTGCTTCG-3′, and the antisense primer for c-KIT was 5’-CCCACGCGGACTATTAAGTCT-3′. The sense primer for CDK7 was 5’-ATGGCTCTGGACGTGAAGTCT-3′, and the antisense primer for CDK7 was 5’-GCGACAATTTGGTTGGTGTTC-3′. The sense primer for OSR1 was 5’- CGGTGCCTATCCACCCTTC-3′, and the antisense primer for OSR1 was 5’- GCAACGCGCTGAAACCATA-3′. The sense primer for GAPDH was 5’-ACCACAGTCCATGCCATCAC-3′, and the antisense primer for GAPDH was 5’-TCCACCACCCTGTTGCTGTA-3′.
